# Modular constraint-based joint dynamic functional brain graph learning model for identifying brain disorders

**DOI:** 10.7717/peerj.21439

**Published:** 2026-09-30

**Authors:** Peiming Xu, Yaru Li, Wei Si, Linmin Wang, Xiao Jiang

**Affiliations:** 1 Shandong Sport University, Jinan, Shandong, China; 2 School of Physical Education, Shandong University, Jinan, Shandong, China; 3 School of Computer and Artificial Intelligence, Shandong Jianzhu University, Jinan, Shandong, China; 4 School of Mathematics Science, Liaocheng University, Liaocheng, Shandong, China

**Keywords:** Functional MRI, Graph learning, Brain atlases, Stochastic block model, Modularity constraint

## Abstract

Resting-state functional magnetic resonance imaging (rs-fMRI) captures spontaneous neural activity and has become a valuable tool for investigating functional alterations in brain disorders. A widely used strategy is to estimate functional connectivity (FC) between brain regions based on blood-oxygen-level-dependent (BOLD) signals, which can be further represented as functional brain graphs (FBGs). Despite promising results, existing methods typically rely on static FBGs derived from a single brain atlas, which limits their ability to capture the temporal variability of brain activity and integrate complementary anatomical information. Moreover, the modular organization of brain networks-an important feature linked to cognitive function and disease-has often been overlooked in model design. To address these limitations, we propose a modularity-constrained dynamic FBG learning framework for brain disorder classification. The proposed method integrates FBGs constructed from multiple brain atlases and captures dynamic spatio-temporal patterns using a sliding window strategy and a Transformer encoder. We employ a stochastic block model (SBM) to detect modular structures, and further incorporate modularity constraints into the training objective to enhance model interpretability and regularize learning. Experiments on the Autism Brain lmaging Data Exchange (ABIDE) and the REST-meta-MDD Consortium (REST-MDD) datasets demonstrate that our method achieves classification accuracies of 68.5% and 65.3%, respectively, outperforming conventional and competitive deep learning methods. This framework provides a novel perspective for dynamic modeling of brain functional connectivity and demonstrates potential for clinical application.

## Introduction

Resting-state functional magnetic resonance imaging (rs-fMRI) is a non-invasive technique that captures spontaneous fluctuations in blood-oxygen-level-dependent (BOLD) signals, offering a window into the brain’s intrinsic functional organization (Khosla et al., 2019; Zhang et al., 2025a; Shon et al., 2026). These signals are commonly used to construct functional brain graphs (FBGs) (Bullmore & Sporns, 2009; Sporns, 2011; Wang et al., 2025), where nodes represent brain regions-of-interest (ROIs) and edges quantify functional connectivity (FC), most commonly estimated using Pearson’s correlation (PC) (Poldrack, 2007; Sporns & Betzel, 2016). Unlike structural brain networks that characterize regional volume, cortical thickness, and the similarity of morphological features across brain regions (Cai et al., 2023), FBGs describe functional interactions between brain regions. FBGs provide a principled framework for characterizing network-level alterations in various neuropsychiatric disorders, including autism spectrum disorder (ASD), major depressive disorder (MDD) (Cohen et al., 2008; Han et al., 2022a), Alzheimer’s disease, and schizophrenia (Bullmore & Sporns, 2009; Fornito, Zalesky & Breakspear, 2015), as they capture whole-brain network interactions beyond isolated voxel- or region-level measurements, thereby enabling a more comprehensive characterization of functional brain alterations.

Recent advances in graph neural networks (GNNs) (Scarselli et al., 2008) and module detection techniques (Poldrack, 2007; Sporns & Betzel, 2016) have enhanced the capacity to extract informative and interpretable features from FBGs. These features hold significant potential for uncovering abnormal network patterns and improving the classification of brain disorders (Fornito, Zalesky & Breakspear, 2015). However, FC is not static; instead, it exhibits temporal variability even during rest. Modern neuroimaging studies show that fMRI-derived brain networks exhibit dynamic interactions between regions. These dynamics are crucial for understanding brain function and the pathological mechanisms of brain disorders (Fornito et al., 2012; Wu et al., 2011; Liu et al., 2017). This highlights the need for learning frameworks that can capture not only the modular structure but also the temporal dynamics of functional brain networks. Recently, Transformer-based architectures have demonstrated success in learning from sequences and dynamic graphs (Bedel et al., 2023; Kim et al., 2024). By attending to all time points in parallel, the self-attention mechanism can effectively capture long-range temporal interactions, offering a promising direction for modeling time-varying brain networks.

Beyond temporal dynamics, another notable limitation of existing methods lies in their reliance on a single brain atlas for graph construction. Although most previous studies have focused on brain graphs constructed from a single parcellation atlas, recent work suggests that different atlases capture complementary aspects of brain networks (Chu et al., 2022). However, integrating information from multiple atlases in a principled way remains an open challenge. Existing multi-view learning methods (Chu et al., 2022; Mahler et al., 2023) often treat atlas-specific graphs as independent views, without fully modeling the shared and distinct patterns across them.

Moreover, a growing body of research highlights the modular architecture of brain networks as a key organizing principle, closely related to cognitive function and neurological disorders (Sporns & Betzel, 2016). The stochastic block model (SBM) (Abbe, 2018; Abbe, Bandeira & Hall, 2016) offers a probabilistic approach for uncovering latent modular structures, yet its integration into end-to-end learning frameworks for neuroimaging tasks remains underexplored. Prior work has either used modularity *post hoc* for interpretation (Arroyo & Levina, 2020) or applied hard module assignments without modeling uncertainty or soft partitions (Paul & Chen, 2020). In addition, recent studies have explored how environmental factors, including air pollution and green space, influence brain structure and function (Liu et al., 2023; Zhang et al., 2025b). These findings contribute to the growing recognition of the complex interaction between environmental exposures and brain health, which could offer additional context for future investigations into brain network alterations.

To simultaneously address these challenges, we propose a modularity-constrained dynamic FBG learning framework for the classification of brain disorders. Specifically, our method incorporates modular structure priors, discovered using SBM, into a deep learning framework to guide functional module learning. To better model brain connectivity, we construct joint FBGs by integrating multiple brain atlases, capturing complementary anatomical information. Moreover, we employ a sliding window mechanism (Lee, Lin & Chen, 2001) to model the temporal dynamics of FC, while a Transformer encoder is used to extract spatio-temporal features from the dynamic graphs. Experimental evaluations on the Autism Brain Imaging Data Exchange (ABIDE) (Di Martino et al., 2014) and the REST-meta-MDD Consortium (REST-MDD) (Yan et al., 2019a) datasets demonstrate that the proposed method consistently outperforms several competitive methods in classifying brain disorders based on fMRI data.

The major contributions of this work are outlined as follows.

- We propose a novel approach for joint brain graph construction by integrating complementary information from multiple anatomical atlases, thereby generating more comprehensive functional brain graphs.
- We address the challenge of modeling the temporal dynamics of FC by introducing a sliding-window strategy coupled with a Transformer encoder for spatio-temporal representation learning.
- We introduce a modular-constrained learning mechanism that incorporates prior knowledge of brain modular structures, derived *via* SBM, into the model’s optimization objective, enhancing both interpretability and classification performance.

The remainder of this article is organized as follows. ‘Related Work’ briefly reviews related research. ‘Materials and Method’ introduces the materials and the proposed method. ‘Results’ presents the experimental setup and results. In ‘Discussion’, we explore the impacts of several key components, Finally, we conclude this article in ‘Conclusion’.

## Related work

### Brain network learning

With the development of deep learning techniques, GNNs have advanced brain network analysis (Kipf & Welling, 2016; Veličković et al., 2017). Graph convolutional networks (GCNs) and graph attention networks (GATs) have been widely used for encoding FBGs and classifying brain diseases (Parisot et al., 2018). For instance, Parisot et al. (2018) leveraged GCNs for neurological disorder classification, and introduced edge-weighted GNNs to better utilize connectivity strength. Despite these advances in GNN-based modeling, most studies rely on single-atlas representations, limiting the ability to capture complementary anatomical and functional information. To address this, recent works have explored multi-atlas learning (Chen et al., 2026; Wang et al., 2022). For example, Chen et al. (2026) proposed an attention-based multi-atlas fusion framework for brain disorder prediction. Inspired by this work, we adopt a multi-atlas learning approach to integrate complementary features from different brain parcellation schemes, thereby enhancing the representational and discriminative capabilities of the model.

### Temporal dynamics learning

Traditional methods estimate static functional networks using PC or partial correlation over the full fMRI time series, yielding one connectivity matrix per subject (Wang et al., 2009; Wang, Zuo & He, 2010; Drysdale et al., 2017). However, they fail to capture the temporal dynamics. Dynamic FC applies a sliding window to segment the time series and build time-varying graphs (Hutchison et al., 2013; Allen et al., 2014), The sliding-window based dynamic FBGs can be viewed as a time-varying system with implicit delays introduced by the windowing mechanism. Similar delay-dependent dynamic behaviors have been extensively analyzed in nonlinear dynamical systems with impulses, underscoring the importance of modeling stable temporal evolution in complex dynamic structures (Liu & Zeng, 2019). However, modeling each window separately limits the ability to capture global temporal dependencies across windows, thereby necessitating learning frameworks that can integrate both short-term dynamics and long-range temporal context.

Recurrent neural networks (RNNs) (Graves, Mohamed & Hinton, 2013) and long short-term memory (LSTM) models (Hochreiter & Schmidhuber, 1997) have been employed to model fMRI dynamics (Yan et al., 2019b), but their inherent sequential processing architecture imposes critical limitations including restricted computational parallelism during training and susceptibility to vanishing or exploding gradients when capturing long-range dependencies. Transformers (Vaswani et al., 2017) offer two key advantages for modeling dynamic brain networks: parallelizable training and a global temporal attention mechanism that effectively captures long-range dependencies. While they have been successfully applied to fMRI sequence learning (Kan et al., 2022; Han et al., 2022b) with improved performance in capturing temporal patterns, existing methods remain primarily based on single-atlas features and may not generalize well to heterogeneous representations from multiple parcellations. Importantly, the joint use of multiple atlases forms a multi-layer or coupled-network representation of the brain, where each atlas provides a structurally distinct yet complementary view. Recent studies have shown that multi-layer coupled network frameworks can enhance representational richness and system-level inference in complex network learning tasks (Fu, Li & Feng, 2025).

### Modularity and topological priors in brain networks

Human brain networks exhibit modular structures, with communities of regions exhibiting stronger intra-module than inter-module connectivity (Sporns & Betzel, 2016). Exploiting such topological priors can enhance representation learning. Several works utilize modularity to regularize or guide brain network learning (Kim, Ye & Kim, 2021). For instance, Kim, Ye & Kim (2021) introduced modularity-aware attention for brain network classification. Leveraged modularity constraints in a matrix factorization framework. However, existing modular approaches often require pre-defined module structures or rely on static brain graphs, limiting their effectiveness on dynamic, heterogeneous networks. To learn modular structures adaptively, SBM (Holland, Laskey & Leinhardt, 1983; Peixoto, 2014) provides a principled probabilistic framework for detecting community assignments and inter-module connectivity patterns. Recent works have explored the incorporation of SBM into deep learning pipelines for structured graph analysis (Duranthon & Zdeborová, 2023), but have rarely been extended to dynamic brain networks or multi-atlas integration.

## Materials and method

### Materials

To validate the effectiveness of the proposed method in the classification of brain disorders, We use two widely used public datasets (*i.e*., ABIDE (http://fcon_1000.projects.nitrc.org/indi/abide) and REST-MDD (http://rfmri.org/REST-meta-MDD)). The original fMRI data from these two datasets undergoes preprocessing. The preprocessing steps include slice-timing correction, motion correction, spatial normalization, and smoothing. These steps aim to eliminate the influence of noise and irrelevant signals and improve data quality.

### Proposed method

The proposed modularity-constrained joint dynamic FBG learning model comprises three key components: (1) multi-atlas FBG construction, (2) spatio-temporal graph representation learning, and (3) modularity-constrained optimization. Figure 1 illustrates the framework of the proposed model.

**Figure 1:**
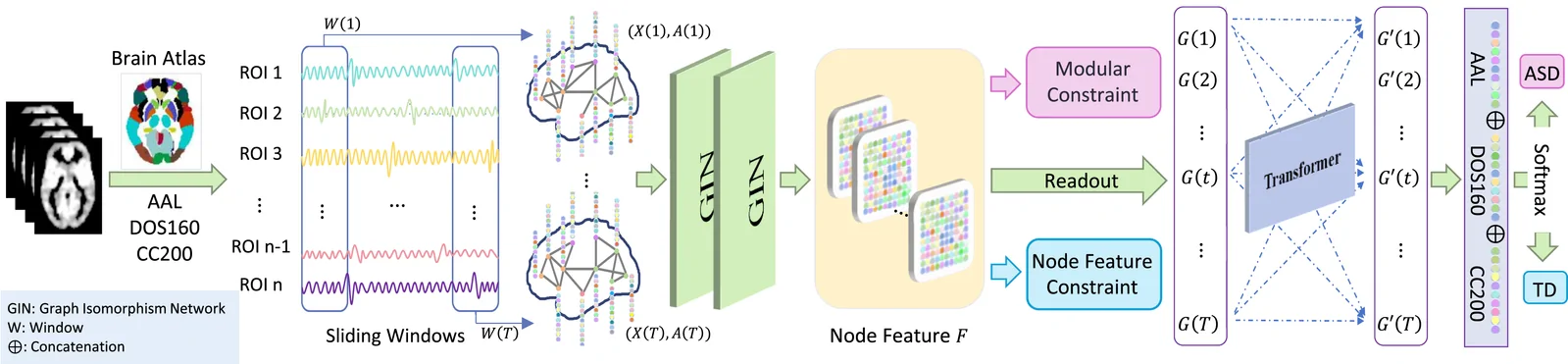
Illustration of the proposed modular constraint-based joint dynamic functional brain graph (FBG) learning model.

#### Multi-atlas FBG construction

The FBGs are constructed based on fMRI data. For each subject, the FBG can be represented as a graph G=(X,A), where *X* denotes the node feature matrix, and *A* represents the adjacency matrix. Considering the dynamic nature of FBGs, a sliding window approach is applied to segment the fMRI time series. The BOLD signals within each time window are used to construct dynamic FBGs. The dynamic FBGs for each subject can be expressed as $G(t) = \left\{ {X(t),A(t)} \right\}_{t = 1}^T$, where t denotes the time window index and *T* is the total number of time windows. We use the PC to calculate the FC strength between brain regions in each time window and employ the derived PC matrix as node feature matrix. The node feature matrices from the AAL, DOS160, and CC200 brain atlases for the t-th time window are denoted as ${X_{AAL}}(t) \in {\mathbb R}^{n_{AAL} \times {n_{AAL}}},{X_{DOS160}}(t) \in {\mathbb R}^{{n_{DOS160}} \times {n_{DOS160}}},{X_{CC200}}(t) \in {{\mathbb R}^{n_{CC200} \times {n_{CC200}}}}$, where n is the number of ROIs.

To remove weak or noisy connections, the adjacency matrices are thresholded. Specifically, only the top 30% of the strongest connections are preserved (*i.e*., the top 30% of the largest values in the node feature matrix are set to 1, while the remaining values are set to 0). This results in sparse FBGs. Given that different brain atlases (*i.e*., AAL, DOS160, and CC200) divide the brain into different sets of ROIs, a joint adjacency matrix *A* is generated for each time window to integrate their complementary features and is denoted as: $A(t) = \left\{ {{A_{AAL}}(t),{A_{DOS160}}(t),{A_{CC200}}(t)} \right\}_{t = 1}^T$.

#### Feature learning for joint dynamic functional brain graphs

To perform dynamic learning on the joint FBGs, we utilize the graph isomorphism network (GIN) (Kim & Ye, 2020; Chen et al., 2019), which is effective at capturing the topological structures of FBGs by aggregating neighboring node features. The formula for GIN is as follows:

(1) $$h_{v}^{(k)} = {{\mathrm{ML}}}{{{\mathrm{P}}}^{(k)}}\left( {(1 + {\varepsilon ^{(k)}}) \cdot h_{v}^{(k - 1)} + \sum\limits_{u{\epsilon}{\scr N}(v)} {h_{u}^{(k - 1)}} } \right),$$

where ${{\mathrm{ML}}}{{{\mathrm{P}}}^{(k)}}$ is a multi-layer perceptron (MLP) at the k-th layer, $h_{v}^{(k)}$ denotes the feature of node v at the k-th layer, ${\varepsilon ^{(k)}}$ is a learnable parameter, and N(v) denotes the set of neighboring nodes of node v. The summation aggregates the features of neighboring nodes u∈N(v), and $(1 + {\varepsilon ^{(k)}})$ adjusts the contribution of the node’s own feature during aggregation. This operation is followed by a non-linear transformation to enhance the model’s expressiveness.

In our model, the adjacency matrix *A* and node feature matrix *X* are used as inputs. Given the dynamic nature of FBGs, the node features at each time window are updated through two GIN layers. After two layers of transformation, the updated node feature matrix *F* is given by:

(2) $$F = f(X,A) = \delta ({\varepsilon ^{(1)}}I + A)[\delta ({\varepsilon ^{(0)}}I + A)X{W^{(0)}}]{W^{(1)}},$$

where δ represents the ReLU activation function which is applied after each layer in the GIN, ${W^{0}}$ and ${W^{1}}$ represent the weight matrices learned in the first and second layers, respectively.

For graph-level classification, the learned node feature matrix F∈F(t) is passed through a readout layer to obtain a graph-level representation. This study adopts an attention-based readout mechanism (Kim, Ye & Kim, 2021), where the attention weights S(t) of the node features are computed as:

(3) $$S(t) = sigmoid({Q^{(1)}}\sigma ({Q^{(0)}}{F_{mean}}(t))),$$

where σ is the nonlinear activation function, ${Q^{(0)}}$ and ${Q^{(1)}}$ are learnable weight matrices, and ${F_{mean}}(t)$ is the vector obtained by averaging the node feature matrix along the node dimension. After obtaining the attention weights S(t), the graph-level representation is:

(4) G(t)=S(t)F(t).

To capture the temporal dynamics of fMRI sequences and analyze the contributions of dynamic FBGs across time, a single-head Transformer encoder (Parmar et al., 2018; Han et al., 2022b) is employed to learn temporal attention. The self-attention mechanism of the Transformer quantifies the significance of different time windows. After Transformer processing, we obtain a new sequence of dynamic graph representations ${G^\prime }(t)$, incorporating both spatial and temporal attention. The final graph-level representation is:

(5) $${G^\prime } = \sum\limits_{t = 1}^T {{G^\prime }} (t).$$

To leverage information from different atlases, we concatenate the representations generated from the AAL, DOS160, and CC200 brain atlases:

(6) $${G_{joint}} = G_{AAL}^\prime \oplus G_{DOS160}^\prime \oplus G_{CC200}^\prime ,$$

where ⊕ denotes the concatenation operation. The joint FBGs with the concatenated features ${G_{joint}}$ are then fed into a fully connected layer followed by a softmax layer for final prediction.

#### Modularity-constrained objective function optimization

To introduce a modular constraint into the objective function, we first employ the SBM for module detection. Using the maximum likelihood estimation method, we calculate the module membership information of nodes and the inter-module connection probability matrix. The specific steps are as follows: Assume there are *K* modules in the network, and each node i belongs to a particular module. Estimate the inter-module connection probability matrix *B*, where ${B_{rs}}$ denotes the connection probability between module r and module s. Solve the model parameters through an optimization algorithm to obtain the module membership information of the nodes. The model employs a novel modular constraint as follow:

(7) $${{\mathscr L}_{mod}} = - \sum _{t = 1}^T\sum _{k = 1}^K\sum _{i,j = 1}^{{N_{k}}}{{{f_{i}^{tk} \cdot f_{j}^{t,k}} \over \left\Vert {f_{i}^{t,k}} \right\Vert \cdot \left\Vert {f_{j}^{t,k}} \right\Vert}},$$

where $f_{i}^{t,k}$ and $f_{j}^{t,k}$ are the embeddings of two nodes in the k-th module (which contains ${N_{k}}$ nodes) at the t-th segment. Such modular constraints are conceptually aligned with block-structured decomposition methods, where structure-preserving algorithms maintain coherent sub-blocks and promote stable representation learning during matrix factorization (Wei et al., 2024). The total modular constraint obtained by summing up the modular constraints calculated using different brain atlases is as follows:

(8) $${{\scr L}_M} = {{\scr L}_{mo{d_{AAL}}}} + {{\scr L}_{mo{d_{DOS160}}}} + {{\scr L}_{mo{d_{CC200}}}}.$$

During training, the cross-entropy loss function ${{\scr L}_C}$ is used to optimize the model parameters. To avoid the problems of gradient vanishing or gradient explosion, an orthogonal constraint (*i.e*., ${{\scr L}_{ortho}} = \left| {|(1/m){F^T}F - I} \right||^{2}$) is added to the node feature matrix. Such orthogonality regularization helps stabilize optimization and maintain the structural integrity of learned representations, which is consistent with structure-preserving orthogonal transformation techniques in matrix computations (Li et al., 2016). The total loss function is therefore formulated as:

(9) $${\scr L} = {{\scr L}_C} + {\lambda _{1}}{{\scr L}_M} + {\lambda _{2}}{{\scr L}_{ortho}},$$

where hyperparameters ${\lambda _{1}}$ and ${\lambda _{2}}$ control the weights of modularity constraint ${{\scr L}_M}$ and orthogonality constraint ${{\scr L}_{ortho}}$ respectively, balancing the contributions between cross-entropy loss ${{\scr L}_C}$ and other regularization terms (Wen & Yin, 2013).

## Results

### Implementation details

The proposed method in this study is implemented on the PyTorch platform and trained using an NVIDIA T600 GPU. The following hyperparameters are set in this study: the network architecture includes two GIN layers and one Transformer encoder layer. For the ABIDE dataset, the window size is set to 40 and the stride to 5; for the REST-MDD dataset, the window size is set to 45 and the stride remains 5. The regularization factors ${\lambda _{1}}$ and ${\lambda _{2}}$ are consistently set to 0.1 for both the ABIDE and REST-MDD datasets. We also discuss the influence of these hyperparameters in a subsequent section. The learning rate is 0.001, the number of training epochs is 40, the batch size is 8, and the dropout rate is 0.5.

### Experimental results and analysis

We use five metrics to comprehensively evaluate the performance of different methods (Genders et al., 2012), including accuracy (ACC), sensitivity (SEN), specificity (SPE) (Swift, Heale & Twycross, 2020), F1-score (F1) (Chicco & Jurman, 2020), and area under the receiver operating characteristic curve (AUC) (Huang & Ling, 2005).

To evaluate the classification performance, we compared our method (denoted as Ours) against a set of baselines, including both classical machine learning techniques and competitive deep learning methods. Specifically, the classical approaches include PC followed by support vector machine classification (PC+SVM) and PC with random forest (Breiman, 2001) (PC+RF), which are commonly used as benchmarks in brain FC analysis. Additionally, we compared against six representative deep learning methods, including GCNs (Kipf & Welling, 2016), identity-aware GCNs (IA-GCNs) (You et al., 2021), GATs (Veličković et al., 2017), neighborhood-enhanced GATs (NE-GATs), dynamic graph modeling (DGM) (Tian, Xiong & Shi, 2021), and brain network Transformer (BNT) (Kan et al., 2022). The settings and implementation details of the comparative experiments are consistent with the configurations reported in Si et al. (2025). The experimental results on the two datasets are shown in Tables 1 and 2, respectively. A t-test is conducted to compare the results of our method with each baseline method, where an asterisk (*) indicates a statistically significant difference ( p<0.05).

**Table 1:** Results of the proposed model and eight different methods on the ABIDE dataset, with best results shown in bold.

Method	ACC (%)	SEN (%)	SPE (%)	F1 (%)	AUC (%)	p-value
PC + SVM	62.2 (4.0)	56.3 (12.0)	66.8 (7.8)	58.4 (9.0)	65.0 (7.6)	0.0013*
PC + RF	61.6 (6.8)	58.9 (13.5)	67.8 (2.0)	65.4 (11.5)	63.0 (7.8)	0.0010*
GCNs	63.7 (7.4)	65.2 (18.4)	59.8 (10.7)	66.8 (13.0)	66.0 (8.1)	0.0235*
IA-GCNs	63.9 (5.2)	61.7 (12.8)	64.8 (5.3)	66.7 (9.0)	60.9 (10.3)	0.0122*
GATs	65.8 (7.6)	59.7 (20.4)	66.9 (14.2)	63.4 (7.6)	67.0 (8.1)	0.0466*
NE-GATs	66.4 (5.8)	57.2 (15.6)	67.4 (5.5)	61.8 (11.6)	66.0 (8.5)	0.0312*
DGM	64.8 (3.5)	64.8 (14.5)	59.6 (9.0)	60.5 (7.3)	64.0 (6.1)	0.0009*
BNT	65.6 (4.6)	**68.2 (11.0)**	57.9 (11.7)	64.1 (6.2)	66.0 (4.5)	0.0242*
Ours	**68.5 (7.1)**	66.4 (8.9)	**68.7 (11.9)**	**68.5 (7.6)**	**74.0 (6.8)**	–

**Note:**

* *p* < 0.05 (two-sample t-test).

**Table 2:** Results of the proposed model and eight different methods on the REST-MDD dataset, with best results shown in bold.

Method	ACC (%)	SEN (%)	SPE (%)	F1 (%)	AUC (%)	p-value
PC + SVM	59.6(8.5)	52.3(18.9)	64.8(19.9)	63.4(12.4)	63.0(12.7)	0.0006*
PC + RF	60.8(5.9)	57.6(9.6)	62.8(3.5)	62.4(5.9)	62.0(7.7)	0.0010*
GCNs	61.7(10.4)	65.2(16.0)	55.8(16.0)	63.8(10.2)	65.0(12.6)	0.0419*
IA-GCNs	61.9(8.6)	60.7(10.3)	58.8(15.4)	66.7(7.5)	65.0(10.7)	0.0290*
GATs	60.8(8.8)	55.7(34.3)	64.8(24.2)	63.4(21.1)	66.0(5.5)	0.0048*
NE-GATs	62.4(4.3)	57.2(22.0)	${\bf{67}}.{\bf{4}}\,({\bf{26}}.{\bf{1}})$	61.8(9.1)	66.0(7.8)	0.0109*
DGM	61.8(12.1)	63.8(15.7)	59.6(23.3)	60.5(10.7)	64.0(13.4)	0.0433*
BNT	61.6(8.3)	65.2(8.7)	56.7(13.2)	64.3(7.4)	67.0(9.9)	0.0216*
Ours	${\bf{65}}.{\bf{3}}\,({\bf{6}}.{\bf{6}})$	${\bf{68}}.{\bf{7}}\,({\bf{15}}.{\bf{6}})$	62.5(17.2)	${\bf{67}}.{\bf{5}}\,({\bf{7}}.{\bf{7}})$	${\bf{70}}.{\bf{0}}\,({\bf{13}}.{\bf{0}})$	–

**Note:**

* *p* < 0.05 (two-sample t-test).

First of all, as shown in Table 1, our method achieves superior classification performance compared to other baseline methods on the ABIDE dataset across most evaluation metrics. In particular, the proposed method (74.0%) achieves an AUC that is 7.0% higher than the second-best method GATs (67.0%). This performance improvement can be attributed to the effective integration of multi-atlas features, which enables the model to capture richer and more robust representations of FC patterns relevant to ASD classification. In addition, Table 2 presents the classification results on the REST-MDD dataset. As we can see, the proposed method (68.7%) achieves an SEN that is 3.5% higher than the second-best method DGM (65.2%). In addition, the proposed method demonstrates competitive performance in terms of ACC, F1-score, and AUC, further highlighting its robustness and generalization ability in distinguishing individuals with MDD from healthy controls. The consistent performance gains observed on both datasets confirm that our joint dynamic FBG learning approach effectively exploits complementary information provided by multiple brain atlases. It can be observed that our method exhibits statistically significant differences compared to other methods, supporting the superiority of our method in brain disease classification. These results underscore the potential applicability of our method in real-world clinical scenarios, offering a promising direction for improving the diagnostic accuracy of brain disorders through advanced graph-based learning frameworks.

## Discussion

### Ablation study

To verify the contribution of each component in the proposed method, we conduct a set of experiments by comparing the proposed method with its five degraded variants, and the results are shown in Tables 3 and 4. We also perform t-test on the outcomes of our method and each degraded variant, and asterisk (*) represents a statistically significant difference ( p<0.05) between our method and a particular method.

**Table 3:** Results of the proposed model and its three variants on the ABIDE dataset, with best results shown in bold.

Model	ACC (%)	SEN (%)	SPE (%)	F1 (%)	AUC (%)	p-value
Ours w/o SBM	63.4 (5.9)	61.8 (22.1)	53.7 (24.6)	62.8 (7.7)	67.0 (3.4)	0.0183*
Ours (AAL-only)	67.1 (10.2)	61.5 (18.5)	68.2 (10.7)	65.4 (11.9)	70.0 (14.9)	0.1065
Ours (DOS160-only)	62.3 (7.4)	51.8 (13.3)	**71.1 (7.0)**	55.8 (10.1)	66.0 (6.6)	0.0274*
Ours (CC200-only)	65.1 (7.2)	59.3 (16.2)	70.4 (10.3)	60.8 (9.4)	71.0 (8.6)	0.0445*
Ours w/o SW	66.8 (9.3)	65.9 (6.9)	63.4 (14.6)	66.1 (8.3)	70.0 (13.7)	0.0360*
Ours	**68.5 (7.1)**	**66.4 (8.9)**	68.7 (11.9)	**68.5 (7.6)**	**74.0 (6.8)**	–

**Notes**:

SBM, stochastic block model; SW, sliding window.

* *p* < 0.05 (two-sample t-test).

**Table 4:** Results of the proposed model and its three variants on the REST-MDD dataset, with best results shown in bold.

Model	ACC (%)	SEN (%)	SPE (%)	F1 (%)	AUC (%)	p-value
Ours w/o SBM	62.4 (6.1)	63.8 (6.8)	54.6 (16.6)	61.9 (3.8)	65.0 (11.4)	0.0247*
Ours (AAL-only)	61.4 (5.1)	63.1 (22.3)	59.3 (21.9)	64.6 (9.2)	64.0 (9.1)	0.0013*
Ours (DOS160-only)	62.1 (9.3)	55.9 (20.6)	**64.8 (26.6)**	63.9 (10.0)	64.0 (15.1)	0.0503
Ours (CC200-only)	63.6 (11.7)	**69.1 (13.7)**	57.8 (23.3)	67.1 (9.5)	66.0 (12.8)	0.0461*
Ours w/o SW	62.8 (8.3)	64.9 (8.7)	59.4 (13.2)	62.1 (7.4)	63.0 (9.9)	0.0194*
Ours	**65.3 (6.6)**	68.7 (15.6)	62.5 (17.2)	**67.5 (7.7)**	**70.0 (13.0)**	–

**Note**:

SBM, stochastic block model; SW, sliding window.

* *p* < 0.05 (two-sample t-test).

As shown in Tables 3 and 4, the ablation study demonstrates the consistent contribution of each proposed component across both the ABIDE and REST-MDD datasets. Firstly, removing the SBM component led to the most significant performance drop, with accuracy decreasing by at least 3% (5% on ABIDE, 3% on REST-MDD), confirming its crucial role in enhancing classification. Secondly, using any single brain atlas (AAL, DOS160, or CC200) instead of the joint multi-atlas information resulted in an accuracy decrease of at least 1.4%. This reduction highlights that integrating complementary information from diverse atlases provides a more comprehensive feature representation, thereby enhancing the model’s performance. Specifically, the AAL atlas achieves the best performance on the ABIDE dataset, whereas the CC200 atlas performs best on the MDD dataset, possibly because different brain parcellation schemes have different impacts on the classification of different brain disorders. Finally, removing the dynamic graph learning module caused a performance decline of at least 1.7%, validating its effectiveness in capturing spatio-temporal dynamics for the classification task.

### Influence of hyperparameter

To further explore the impact of key hyperparameters in the proposed method on classification performance, we conduct a comprehensive analysis, including the number of modules *K*, sliding window size *L*, and the constraint weights ${\lambda _{1}}$ and ${\lambda _{2}}$. The experimental results are shown in Figs. 2, 3, and 4, respectively.

(1) **Number of modules *K*.** As illustrated in Fig. 2, on the ABIDE dataset, *K* was varied from 2 to 6. The model achieved the highest classification accuracy of 68.5% when *K* was set to 3. On the REST-MDD dataset, the same range of *K* was tested, and the best performance (65.3%) was obtained when *K* was set to 4. These results indicate that the number of modules significantly affects model performance. Too few modules may fail to effectively capture the modular structure of FBGs, resulting in insufficient feature representation. Conversely, too many modules may introduce redundant information, increase model complexity, and potentially lead to overfitting. Therefore, selecting an appropriate number of modules is essential for achieving optimal classification performance.
(2) **Sliding window size *L*.** Figure 3 shows the impact of sliding window size on model performance. For the ABIDE dataset, the tested values of *L* range from 30 to 50. The model achieves the highest classification performance of 68.5% when *L* is set to 40. For the REST-MDD dataset, the tested values of *L* range from 35 to 55, and the model reaches the highest accuracy of 65.3% when *L* was set to 45. These findings demonstrate that the choice of sliding window size is a crucial factor influencing the model’s performance. A window that is too small may fail to capture sufficient temporal information, leading to incomplete feature representations. On the other hand, an overly large window may introduce redundant information, increase computational burden, and increase the risk of overfitting, thus impairing the model’s generalization ability. Therefore, a properly selected sliding window size facilitates the extraction of dynamic FC features and improves classification accuracy.
(3) **Constraint weights ${\lambda _{1}}$ and ${\lambda _{2}}$.** As shown in Fig. 4A, on the ABIDE dataset, when ${\lambda _{1}}$ is set to 0.1 and ${\lambda _{2}}$ to 0.1, the model achieves the best balance between classification performance and training stability, resulting in an accuracy of 68.5%. Under the same settings, the proposed model attains an accuracy of 65.3% on the REST-MDD dataset, as shown in Fig. 4B. On the ABIDE dataset, further analysis shows that when ${\lambda _{2}}$ is fixed and ${\lambda _{1}}$ is varied, the classification accuracy shows larger fluctuations compared to when ${\lambda _{1}}$ is fixed and ${\lambda _{2}}$ is varied. This suggests that the model is more sensitive to changes in ${\lambda _{1}}$. In contrast, variations in ${\lambda _{2}}$ cause relatively smaller performance fluctuations, indicating that the model is less sensitive to this parameter. Specifically, increasing ${\lambda _{1}}$ leads to a decline in classification accuracy, which may be attributed to over-smoothing effects from overly strong modular constraints. Increasing ${\lambda _{2}}$ while keeping ${\lambda _{1}}$ fixed also results in decreased accuracy, likely due to the strong orthogonality constraint on the node feature matrix hindering model convergence and degrading performance. Similar trends can also be observed on the REST-MDD dataset. Hence, appropriate tuning of ${\lambda _{1}}$ and ${\lambda _{2}}$ is critical for effective model training and performance optimization.

**Figure 2:**
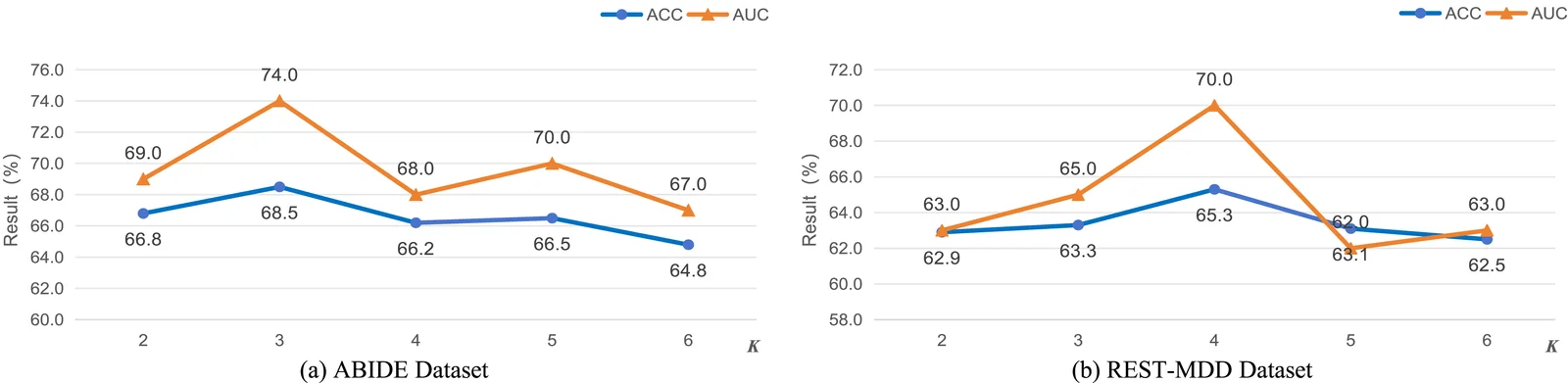
Effect of module number (*K*) on model performance for ABIDE and REST-MDD datasets.

**Figure 3:**
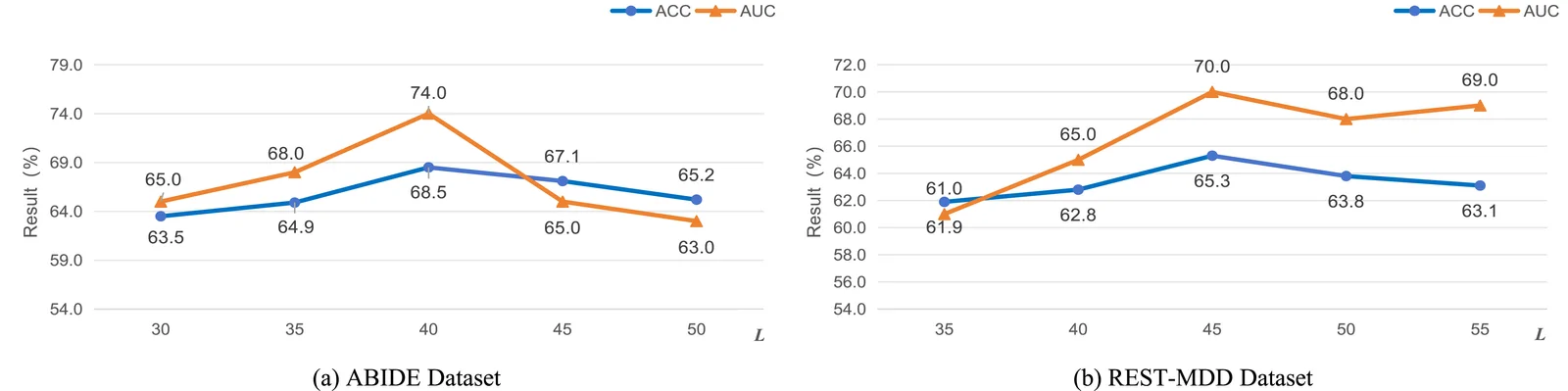
Effect of the length of sliding window (*L*) on model performance for ABIDE and REST-MDD datasets.

**Figure 4:**
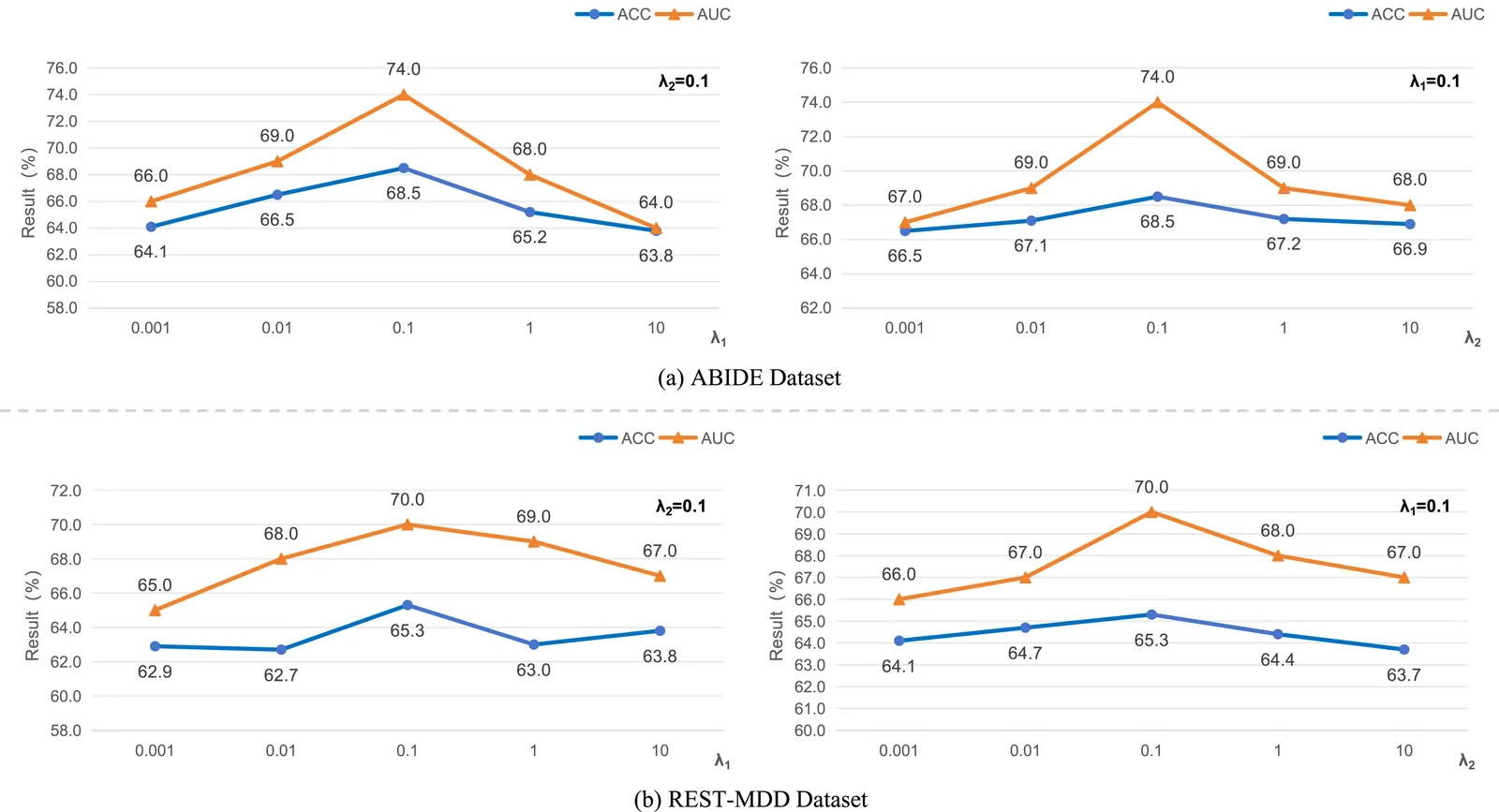
Influence of constraint weights ( ${\lambda _{1}}$ and ${\lambda _{2}}$) on results for the ABIDE and REST-MDD datasets.

### Visualization analysis

We input rs-fMRI data from both typically developing (TD) individuals and patients into the proposed model and visualize the resulting FBGs for comparison. The results are presented in Fig. 5. Specifically, the FBGs of the TD control group are compared separately with those of both ASD and MDD patient groups. To more intuitively present the FBGs constructed after modular feature extraction, the visualization tool BrainNet Viewer (Xia, Wang & He, 2013) is employed, and visualizations are rendered from three different perspectives.

**Figure 5:**
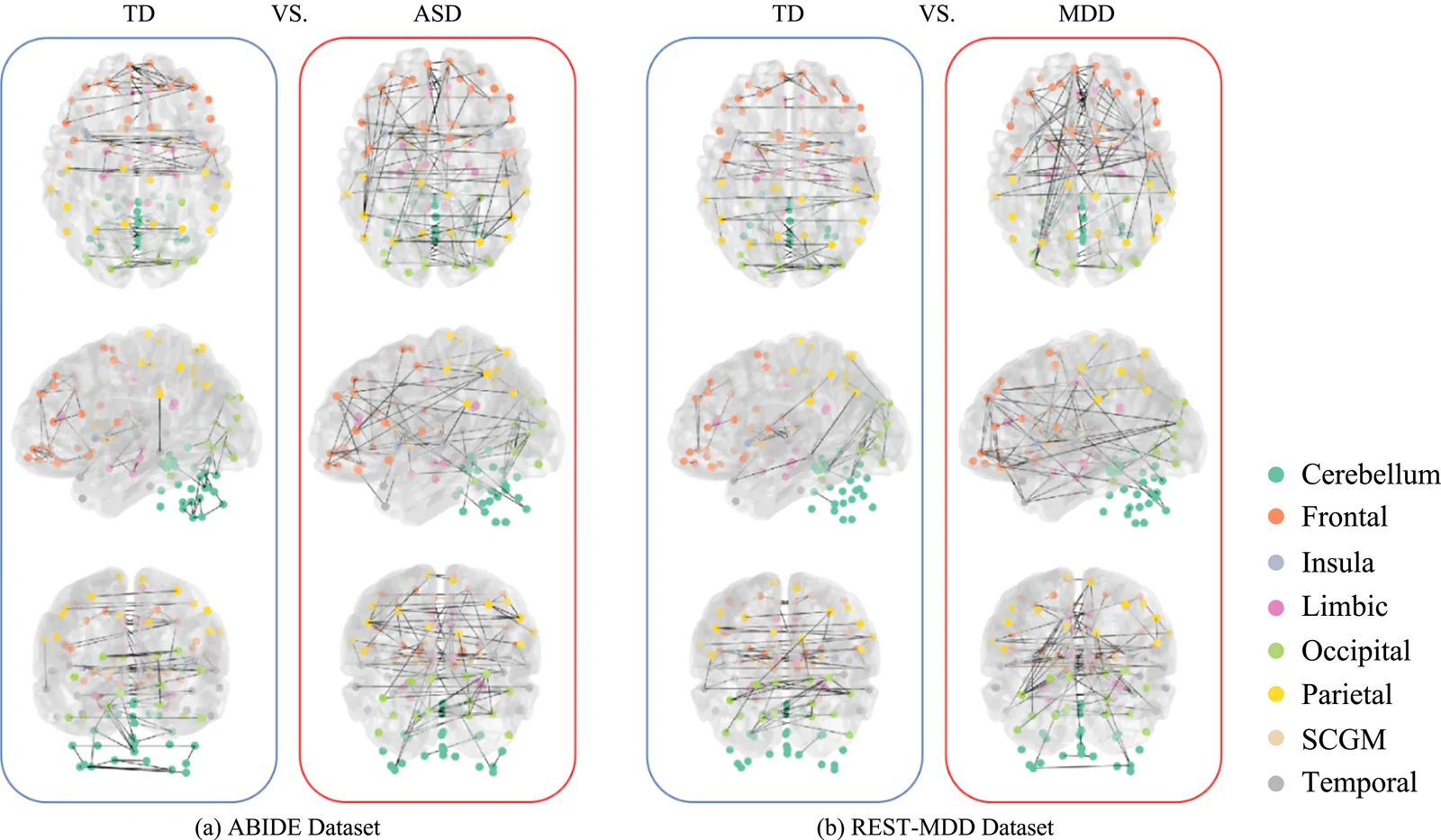
Comparison between typically developing (TD) controls and patients on the ABIDE and REST-MDD datasets.

As shown in Fig. 5A, dense intra-regional functional connections are observed within the frontal and parietal lobes in the TD control group, particularly visible in the axial view, while ASD patients show increased inter-regional and long-range connectivity, suggesting more complex network patterns. These abnormal connections may be related to social and behavioral deficits in ASD (Li et al., 2013; Zhang et al., 2018). As shown in Fig. 5B, abnormal long-range connectivity is observed between the orbitofrontal cortex and occipital lobe, as well as between frontal and parietal regions. These disruptions are linked to impairments in visual processing, language, and cognitive control (Liu et al., 2024; Helm et al., 2018; Kwaasteniet et al., 2013). Overall, the results reveal distinct connectivity patterns between patient groups and TD control group. The integration of modularity improves both classification accuracy and the interpretability of the model, offering insights into the neural mechanisms of ASD and MDD.

## Conclusion

In this study, we propose a modularity-constrained joint dynamic FBG learning model to improve the accuracy and enhance the interpretability of brain disorder classification. By integrating multiple brain atlases, the proposed model constructs joint FBGs, thereby enriching feature representation. To capture spatio-temporal dynamics, we employ the sliding window mechanism coupled with a Transformer encoder, while a SBM is applied to detect modular structures, thus introducing modular constraints that guide the learning process. Experiments on two datasets demonstrate the effectiveness of our model, and ablation studies confirm the benefits of both multi-atlas integration and modular constraints. Visualization results further reveal significant FBG differences between TD individuals and patients, highlighting the model’s potential for neuroscience and brain disorder research. In future work, we aim to incorporate multimodal data, such as electroencephalography (EEG) (Yeh et al., 2024; Ye et al., 2024; Pei et al., 2025), to capture richer features and thereby improve the performance of brain disorder identification.

## Additional Information and Declarations

### Data Availability

The following information was supplied regarding data availability:

The Autism Brain Imaging Data Exchange Data is available at ABIDE: https://fcon_1000.projects.nitrc.org/indi/abide/abide_I.html.

The REST-meta-MDD data is available at The R-fMRI Network: http://rfmri.org/REST-meta-MDD.

The code is available at GitHub and Zenodo:

- https://github.com/BNA278/ModularFBGs.

- BNA278. (2026). BNA278/ModularFBGs: ModularFBGs (v1.0). Zenodo. https://doi.org/10.5281/zenodo.19759730.
